# What a Difference a Stochastic Process Makes: Epidemiological-Based Real Options Models of Optimal Treatment of Disease

**DOI:** 10.1007/s10640-017-0168-x

**Published:** 2017-06-21

**Authors:** C. E. Dangerfield, A. E. Whalley, N. Hanley, C. A. Gilligan

**Affiliations:** 1 Department of Plant Sciences, University of Cambridge, Downing Street, Cambridge, CB2 3EA UK; 2 Warwick Business School, University of Warwick, Coventry, CV4 7AL UK; 3 School of Geography and Geosciences, Irvine Building, University of St Andrews, North Street, St Andrews, Fife KY16 9AL UK

**Keywords:** Real options, Logistic SDE, Disease control, Stochastic epidemics, Optimal timing

## Abstract

The real options approach has been used within environmental economics to investigate the impact of uncertainty on the optimal timing of control measures to minimise the impacts of invasive species, including pests and diseases. Previous studies typically model the growth in infected area using geometric Brownian motion (GBM). The advantage of this simple approach is that it allows for closed form solutions. However, such a process does not capture the mechanisms underlying the spread of infection. In particular the GBM assumption does not respect the natural upper boundary of the system, which is determined by the maximum size of the host species, nor the deceleration in the rate of infection as this boundary is approached. We show how the stochastic process describing the growth in infected area can be derived from the characteristics of the spread of infection. If the model used does not appropriately capture uncertainty in infection dynamics, then the excessive delay before treatment implies that the full value of the option to treat is not realised. Indeed, when uncertainty is high or the disease is fast spreading, ignoring the mechanisms of infection spread can lead to control never being deployed. Thus the results presented here have important implications for the way in which the real options approach is applied to determine optimal timing of disease control given uncertainty in future disease progression.

## Introduction

Invasive pests and pathogens are increasing worldwide, posing a threat to agriculture and forestry production (Gilligan 2008; Strange and Scott 2005). Deciding whether or not to implement expensive control strategies is complicated by uncertainties about the future spread of a pest or pathogen, and by the irreversible nature of many control strategies (felling a forest stand, for example). Therefore it may be best not to apply treatment immediately, but to wait and see how the disease progresses (Sims and Finnoff 2013), partly because learning may occur over time about the spread and impacts of the disease and about the effectiveness of alternative control strategies.

The real options approach has been used to investigate the impact of uncertainty on the timing of control actions for disease outbreaks, as it provides a convenient way to couple uncertainty with economic analysis (Sims and Finnoff 2013). Viewing disease control as an option which can be exercised to reduce current and future damages to the host species of a pest or pathogen, the real options framework can be used to determine the optimal timing of control (Ndeffo Mbah 2010; Saphores 2000). That is, when is it optimal to use the option to control the disease? How long should we delay actions in order to learn more? This paper advances the discussion by incorporating epidemiological models of disease spread within a real options format. As we show, sacrificing epidemiological accuracy for computational ease can significantly change the management recommendations which emerge in terms of when it is best to intervene.

To incorporate uncertainty into the decision making process, the progress in the level of infection is described by a stochastic process. Traditionally, the real options approach assumes the change in the infected population follows a geometric Brownian motion (GBM) (Saphores 2000; Sims and Finnoff 2012). The advantage of such a simplifying assumption is that GBM is well-understood and allows for easily interpretable, closed-from solutions to the real options problem. However, using the GBM process essentially assumes that the mean level of infection grows exponentially, and while this holds for the early part of the epidemic, it does not capture the longer term limiting behaviour effected by the finite number of susceptible hosts.

The finite number of susceptible hosts, which could represent a forest area or agricultural field, impacts the evolution of the epidemic in two ways. Firstly, there is a natural upper boundary for the system, namely the size of the susceptible population, and so the level of infection must remain below this boundary for solutions to make sense. Secondly, there is a deceleration in the rate of infection as this upper boundary is approached, which occurs due to the lack of susceptibles that can be infected (Large et al. 1946). Geometric Brownian motion does not capture either of these two key characteristics of disease spread, since the process is unbounded and furthermore assumes the mean level of infection grows exponentially even when the level of infection is large. Therefore, GBM is an unrealistic description of the increase in infection level for many diseases and pests.

Epidemiological modelling uses mathematical models to describe the evolution in the level of infection within the host population based on the characteristics of infection spread (Keeling and Rohani 2008; Gilligan and van den Bosch 2008). The level of infection increases when there is a contact between an individual capable of spreading infection with an individual not yet infected (i.e. they are susceptible to infection), such that transmission occurs. This contact may not be physical but could for example be proximity to an infectious individual, as is the case with wind-borne diseases such as ash dieback (Gross et al. 2014). Epidemiological models explicitly capture this mode of transmission by assuming that the rate of change in the level of infection depends on interactions between the susceptible and infectious populations, and so depends on the current level of susceptible individuals (Keeling and Rohani 2008; Gilligan and van den Bosch 2008). This has the effect of accelerating the rate of infection when the proportion of susceptible hosts is high and decelerating it when the proportion is low (Gilligan 2008; Keeling and Rohani 2008). In deterministic formulations of such models the evolution in the level of infection over time is described by a logistic-type term, which not only ensures that the level of infection respects the natural upper boundary of the system but also that the rate of growth in infection level slows down as the upper boundary is approached. This logistic-type growth in infection level is consistent with results from experimental studies (Large et al. 1946) and so is more biologically accurate than the assumption of exponential growth. Epidemiological modelling frameworks are well established and have become an important tool in understanding the invasion and persistence of pathogens (Gilligan and van den Bosch 2008) as well as in the design of control strategies within human, animal and plant health. For example, epidemiological models were instrumental in the implementation of the HPV vaccination program within the UK (Choi et al. 2010). In animal health, epidemiological models have also been used to investigate the impact of different control measures to combat the rise in bovine tuberculosis cases in the British cattle industry (Brooks-Pollock et al. 2014). Finally within plant health they have been used to assess the efficacy of control measures for agricultural diseases, such as rhizomania of sugar beet (Gilligan et al. 2007) as well as tree diseases such as Bahai bark scaling in north-eastern Brazil (Cunniffe et al. 2014) and sudden oak death in California (Cunniffe et al. 2016). However, to date such modelling approaches have been largely ignored within the real options literature on the optimal timing of disease control.

The principal objective of this paper is therefore to show how epidemiological-based modelling approaches can be used within the real options framework; and to illustrate the impact these approaches have relative to the standard GBM approach. By contrasting the standard approach (GBM) with two alternative, more realistic, models for infection dynamics, we show that using an inappropriate stochastic process to describe uncertainty in the level of infection leads to sub-optimal timing of control measures. The first alternative model incorporates logistic-type behaviour into the drift term, but has the same diffusion term as GBM and we call this the mean-reverting (MR) model. The second model incorporates a logistic-type noise term into the diffusion term as well as the drift term and is termed the logistic model. Both alternative formulations to GBM arise directly from the *SI*-type (Suscetptible-Infected) epidemiological model, according to different assumptions associated with the incorporation of the noise term into the deterministic equation. An added advantage of the logistic SDE is that respects the natural upper boundary of the system, whereas the MR model does not. Therefore, the logistic SDE is the only model of the three studied here that captures both characteristics of disease spread outlined above.

A key aim of this paper is to examine the sensitivity of the timing of disease control to the assumptions regarding the uncertainty in the future level of infection. By using a simple decision problem we demonstrate how the differences between the stochastic processes alone affect the optimal timing for disease control. It also facilitates comparison with well-known results for GBM. Furthermore, since incorporating more complicated SDEs for the infection dynamics means solutions must be obtained numerically, focussing on a very simple decision problem ensures numerical simulations remain tractable.

Our approach is general, but for illustration we focus on the optimal timing of a one-off application of treatment to a single species forest stand or agricultural monoculture. We motivate our analysis by considering systems in which a single application of a fungicide or release of a biocontrol agent can prevent economic loss in value by reducing infection. For example, within the forestry context, stump treatment for *Heterobasidion annosum*,^1^ an economically important disease within commercial conifer forestry in the UK, can effectively stop infection on freshly cut stumps and so prevents disease damage to the timber (Nicolotti et al. 1999). Another example is the release of *Rhizophagous grandis* as a biological control against the spruce bark beetle *Dendroxonus micans*.^2^ *R. grandis* was introduced in the 1980’s in Britain by the Forestry Commission and it is now successfully established so that levels of the bark beetle are very low and do not cause significant damage to timber (Fielding et al. 1997).

Our results suggest that ignoring either of the two key characteristics of disease spread (logistic-type growth; an upper limit) leads to over-valuation of the option to treat, and thus to suboptimally postponed deployment of control. Furthermore, in certain situations (high volatility and/or fast spreading infection) the GBM and MR models result in control never being deployed, leading to losses due to disease damage. We quantify the loss in value if treatment is suboptimally postponed and show that the proportional loss could be substantial, particularly for high volatility and/or fast spreading infection. This critical difference between the GBM and MR models and the logistic model arises due to the fact that the GBM and MR models over-estimate the value that can be obtained from applying treatment since they allow the potential level of infection to become unrealistically large. Our results highlight the dependence of the conclusions from the real options model on the form of the stochastic process used to describe the uncertainty in the future progress of infection. Therefore it is important to ensure that this process accurately captures the key characteristics of disease spread. We argue that the logistic SDE is the only one of the three models considered here that captures these.

The structure of the remainder of this paper is as follows. In Sect. 2 we review the literature on control of invasive pests and pathogens. Section 3 motivates the new stochastic differential equations (SDEs) proposed to model the spread of infection and derives the associated real options model. The results are presented in Sect. 4 and finally Sect. 5 discusses the implications of the results and concludes.

## Literature Review

The real options approach has been used within the environmental economics literature to incorporate ecological uncertainty into the valuation of management options of renewable resources such as forests (Insley and Rollins 2005; Insley and Lei 2007) as well as in agriculture (Koppl and Koppl-Tuyna 2013; Tzouramani and Mattas 2004). Here, we focus on the control of invasive pests and pathogens, since the main application of the model presented in this paper is to determine the deployment of treatment to minimise the expected net costs of damage due to an epidemic outbreak. Given the ecological uncertainty that characterises the future spread of infection, and the irreversibility of some actions that can be taken in response to such a risk, then a real options approach is attractive. The first application of real options to pests and diseases by Saphores (2000) considered the optimal timing of pesticide application under future uncertainty in a pest population. Varying the level of uncertainty in the pest population dynamics, Saphores (2000) showed that greater uncertainty in future pest densities increases the threshold pest density at which it is optimal to spray, since the probability that the pest population will become small is larger. The analysis gives rise to the “wait and see” approach in dealing with invasive species; the idea being that when there is great uncertainty in the future dynamics of the invasive species, there is value in waiting to learn more before investing in control.

Sims and Finnoff (2012, 2013) have since extended this initial work in a number of ways. Sims and Finnoff (2013) consider the implications of the reversibility of the control strategy on (i) how long a regulator should wait to take action and (ii) the severity of the action taken (i.e. the extent of control measure). They find that if control measures are partly reversible (for example trade bans), then it is optimal always to act as soon as possible, i.e. never to adopt a wait-and-see policy. This emphasises the fact that reversibility of actions is key, and that there is a trade-off between the speed and the severity (i.e. reversibility) of actions taken. Sims and Finnoff (2012) consider the impact of a spatial boundary on timing of control by treating the maximum area that can be affected as an upper limit within the real options framework. They find that this spatial scale can impact the timing and stringency of control strategies, and so incorporating an upper bound is important in planning measures to minimise losses from infection and disease damage (Sims and Finnoff 2012).

The real options approach has also been used to investigate the timing of specific control measures to minimise net damage costs from disease or invasive species. Sims (2011) considers the optimal timing of salvage harvest to recoup timber values following a disease outbreak in a forest crop, and finds that slower rates of forest growth delay the optimal timing of salvage harvest, while large timber and non-timber values suggest more immediate action is optimal. Multiple interacting control options, namely chemical and biological control, are considered in Marten and Moore (2011). They find that biological control is sufficient to manage the pest so long as infestation can be detected and controlled without substantial delay. However, if the pest reaches high levels before controls can be employed a more costly combined strategy involving biological and other methods is optimal for pest management (Marten and Moore 2011).

In most studies, the economic value of the damage caused by the disease or pest is the main focus, and the aim of the control is to minimise the damage cost within the real options framework. Ndeffo Mbah (2010) instead consider the value added by applying control (namely treatment for a disease or pest) in terms of the monetary gain per unit of infection treated. Furthermore they incorporate a logistic term into the drift coefficient of the stochastic process describing the spread of infection, and so the mean growth of the process is limited by a parameter that represents the carrying capacity. In particular they find the new SDE leads to a difference in the optimal time to treat when compared with the standard GBM assumption. The difference highlights the dependence of the real options approach on the formulation of the underlying model for uncertainty in the disease dynamics. However, the disadvantage of the approach taken in Ndeffo Mbah (2010) is that the stochastic process can increase above the carrying capacity (Sarkar 2009). While in certain applications (e.g. harvesting fish) it is reasonable for the population to increase above the carrying capacity, in the study of disease spread this parameter represents a physical limit such as the finite number of trees or plants within a fixed area that can be infected. Therefore it is impossible for the area infected to reach a value greater than this bound and so trajectories of the stochastic process that go beyond this point do not have any applicable meaning in this context.

Although the impact of uncertainty in disease spread on the optimal timing of control measures has been studied within the economics literature, there has been an artificial separation between traditional epidemiological models and those used within the real options framework. A significant contribution of this paper is to show how the uncertainty in disease spread can be formulated directly from basic epidemiological principles for incorporation and analysis in the real options framework. This leads to two different SDEs: the first of which has previously been studied in Ndeffo Mbah (2010) and the second of which incorporates a logistic-type term into the diffusion as well as the drift coefficient. To the best of our knowledge this is the first time that such a process has been used within the real options framework to determine the optimal timing of control. The complexity of this new stochastic process means that the real options model no longer permits closed form solutions. We frame the problem in a similar manner to Ndeffo Mbah (2010) by assuming that treatment eradicates infection, and so damage due to disease is not permanent (and so in the forestry or agricultural context, once trees or crops have been treated there is no loss in timber or crop value in future time periods).

## Real Options Model

### Setup

Consider a disease outbreak in a particular crop or tree species within an area of fixed size, which could for example be a forest stand, an agricultural field or a plantation. In particular, we consider that the number of trees or other plant hosts remains fixed. This is often the case in agriculture or even-aged forest stands where typically crops or trees are planted and then harvested at some future time period. We assume that there is a control available that would remove current damage caused by infection which can be applied at any time for a one-off fixed cost, and this treatment is completely irreversible. Note that the level of infection may remain at some low endemic level post treatment, but we assume that such a level is low enough so as not to cause any significant damage to the economic return that can be expected from the timber or crop. Furthermore there is uncertainty in the future levels of infection due to environmental and demographic noise associated with the transmission process for infection. The decision-maker is faced with the following choice: should treatment be administered immediately or should the decision-maker wait to learn more about the progression of the epidemic? Waiting allows the decision-maker to determine whether the level of infection gets worse or better over time.

Traditional net-present-value (NPV) analysis would advocate undertaking treatment providing the value of the investment (i.e. the application of treatment and the associated savings in economic losses), is greater than the cost, . However, due to uncertainty in disease dynamics combined with the irreversibility of the decision to treat, there is value in delaying treatment so as to learn more about the progress of the disease (Dixit and Pindyck 1994). That is, there is a value associated with the option to treat.

To include uncertainty into the decision making approach, we assume that the level of infection, *I*, can be described by a stochastic process. Traditionally the increase in the level of infection is assumed to follow geometric Brownian motion (GBM) and so the dynamics of the level of infection is given by the following GBM SDE (Saphores 2000; Sims and Finnoff 2012, 2013)

1 $$\begin{aligned} \hbox {GBM } \, {\text {d}}I = {\upbeta }I{\text {d}}t + {\upsigma }I{\text {d}}W, \end{aligned}$$

where $\upbeta$ is the rate of transmission of infection, $\upsigma$, which we term the volatility, is a parameter that scales the amount of uncertainty, *I* is the current level of infection and $\hbox {d}W$ is a Wiener increment. The advantage of using the GBM is that the logarithm of the infected area follows a Brownian motion, and so analytic solutions to the real options model can be obtained. However, under this assumption the level of infection is unbounded and so trajectories of the process can violate the natural upper boundary of the system, namely the finite, fixed number of susceptible individuals. Furthermore, GBM assumes that the mean level of infection grows exponentially. While such an assumption is arguably a good approximation in the early stages of the epidemic, it does not capture the slowdown in the rate of infection as the level of infection becomes large due to the limited number of susceptible individuals. Therefore, it is not sufficient to simply enforce an upper boundary on GBM, since the process does not accurately capture the logistic-type behaviour in infection growth, which is characteristic of a pathogen spreading through a finite population.

### Epidemiologically-Based Model of Uncertainty in Disease Spread

Instead of assuming the level of infection follows a given stochastic process, we derive a stochastic model for the change in the level of infection over time based on the mechanisms of disease spread. The population is compartmentalised based on their infection status, and we assume that there are only two infection states; susceptible to infection (*S*) or infected and able to transmit infection (*I*). This is known as the Susceptible-Infected (*SI*) model within the epidemiological literature (Keeling and Rohani 2008). The increase in the number of infected individuals is given by the product of the per capita rate at which a susceptible host contracts infection times the number of susceptible individuals. The rate at which a susceptible contracts infection is, in turn, given by the rate of transmission per infected contact, $\upbeta$, times the probability of contact with an infectious individual, $I/I_{\mathrm{max}}$, where $I_{\mathrm{max}}$ is the maximum number of potential infected individuals. We note that the parameter $\upbeta$ captures both the probability of transmission and the contact rate. Assuming the total population remains constant, $\hbox {I}_{\mathrm{max}}$ is equivalent to the total population size and so $=I_{\mathrm{max}}-I$. Initially, ignoring uncertainty in disease spread, the evolution in the level of infection is therefore given by the following ordinary differential equation (ODE),

$$\begin{aligned} \frac{\hbox {d}I}{\hbox {d}t}={\upbeta }I\left( {1-\frac{I}{I_{\mathrm{max}} }} \right) . \end{aligned}$$

This *SI* model is typical for a number of plant pathogens since frequently plants do not recover from disease. For example the SI model was used to describe the spread of sudden oak death in mixed species stands (Ndeffo Mbah and Gilligan 2010).

Uncertainty in disease spread is incorporated by assuming there is variability in the transmission parameter, ${\upbeta }$, driven by external forces. For example, fluctuations in temperature and climate have been shown to modify the infection rate (Sturrock et al. 2011). Here we assume that the fluctuations in transmission rate are stationary. There are two different approaches to incorporating this form of variation.

Firstly the ‘corrected apparent infection rate’ is perturbed, leading to ${\upbeta }\left( {1-I/I_{\mathrm{max}} } \right) \rightarrow {\upbeta }\left( {1-I/I_{\mathrm{max}} } \right) \,+\,\upsigma \upxi$ (Marcus 1991), where ${\upxi }$ is white noise and ${\upsigma }$ is a constant that controls the magnitude of the perturbation. The uncertain evolution of future disease spread is described by the following mean-reverting (MR) SDE,

2 $$\begin{aligned} \hbox {Mean-Reverting (MR) }\quad \hbox {d}I={\upbeta }I\left( {1-\frac{I}{I_{\mathrm{max}} }} \right) \hbox { d}t+{\upsigma }I\hbox {d}W. \end{aligned}$$

The MR SDE in Eq. (2) has been used within the real options framework in previous studies to describe the increase in infected area (Ndeffo Mbah 2010) as well as the growth in pest populations (Marten and Moore 2011). When the level of infection reaches $I_{\mathrm{max}}$, the magnitude of the diffusion term is non-zero and so there is a positive probability that the level of infection will exceed $I_{\mathrm{max}}$, which is unrealistic for a fixed host population and questions the applicability of this stochastic process to the problem at hand.

Alternatively the transmission rate itself is perturbed, leading to ${\upbeta }\rightarrow {\upbeta }+{\upsigma \upxi }$ and so the evolution in the level of infection is given by the following logistic SDE,

3 $$\begin{aligned} \hbox {Logistic }\quad \hbox {d}I={\upbeta }I\left( {1-\frac{I}{I_{\mathrm{max}} }} \right) \hbox {d}t+{\upsigma }I\left( {1-\frac{I}{I_{\mathrm{max}} }} \right) \hbox {d}W. \end{aligned}$$

As the level of infection, *I*, reaches $I_{\mathrm{max}}$, both the drift and diffusion term approach 0 and so the trajectories of the SDE remain within the interval $\left[ {0,\,I_{\mathrm{max}} } \right]$. Therefore the physical upper boundary of the total population size is preserved directly within the dynamics of the logistic SDE.

The logistic SDE approach provides a way of relating the uncertainty in future levels of infection to the randomness of the transmission process due to environmental factors. Therefore it provides an epidemiological-based approach to incorporating uncertainty into the decision problem. Furthermore this approach can be extended to more complex epidemiological models, for example in the case of diseases where there is an additional recovery state (termed the $\hbox {SIR}$ model).

### The Decision Problem

We assume that the effect of treatment is to eradicate current and future damages as a result of infection and, for simplicity, we assume treatment is applied instantaneously. Examples of such treatment within a forestry context are, stump treatment for *H. annosum*,^3^ and the fungal biocontrol agent *R. grandis* to control *D. micans*,^4^ a spruce bark beetle (see introduction for details).

We only consider the gain in economic value from the timber or crop saved and not from the wider environmental damage that may be reduced. Therefore the value of applying treatment is simply:

4 $$\begin{aligned} V_t =pI_t, \end{aligned}$$

where *p* is the gain in yield per unit of infected area treated, which is assumed to be constant over time and the level of infection, $I_t$, varies stochastically over time according to Eqs. 1, 2 or 3. This form of the value function is similar to that used in Ndeffo Mbah (2010).

While more complicated formulations of the value from disease control have previously been used (Saphores 2000; Sims and Finnoff 2012), here we focus on a simple decision problem since the main focus of this paper is the comparison amongst the different stochastic processes rather than the complexity of the decision problem itself.

Viewing the application of treatment as an investment with value $V_t$, the decision problem can be viewed as a real option (Dixit and Pindyck 1994), which analogously to a financial option (Black and Scholes 1973) is the right but not the obligation to make an investment for a fixed price in the future. The payoff from applying treatment at time *t* is $V_t -C$ and so we want to maximise the expected present value,

5 $$\begin{aligned} F\left( V \right) =\max \mathbb {E}\left[ {\left( {V_{t_*} -C} \right) e^{-rt_*}} \right] . \end{aligned}$$

Here $t_*$ is the time in the future at which the decision is made, $\hbox {r}$ is the discount rate and $\mathbb {E}$ denotes the expectation. The expectation must be taken since $I_t$ (and therefore also $V_t$) is a stochastic process. This is an optimal stopping problem, and so we must find the threshold at which the value from applying treatment immediately is maximal. Using standard methods from dynamic programming, the value of the option to apply treatment, *F*(*V*), must satisfy the following Bellman equation (Dixit and Pindyck 1994) (see “Appendix” for details)

6 $$\begin{aligned} \frac{1}{2}b\left( V \right) ^{2}\frac{d^{2}F}{dV^{2}}+a\left( V \right) \frac{dF}{dV}-rF=0. \end{aligned}$$

The functions $a\left( V \right)$ and $b\left( V \right)$ for each stochastic process used to describe the level of infection are given in Table 1.

$$\begin{aligned}&\displaystyle F\left( 0 \right) =0\\&\displaystyle F\left( {V^{*}} \right) =V^{*}-C\\&\displaystyle \frac{\hbox {d}}{\hbox {d}V}F\left( {V^{*}} \right) =1. \end{aligned}$$

$V^{*}$ is the value at which treatment should be applied immediately. It represents the boundary between the *continuation region* and the *exercise region* (region in which treatment is applied). The first condition follows from the fact that if the value of applying treatment goes to 0 it remains at 0, i.e. infection cannot be re-introduced from an outside source. The second condition is called the *value matching condition* (Dixit and Pindyck 1994), which states that when treatment is undertaken immediately the option value equals the net gain of $V^{*}-C$. Finally the last condition is the *smooth pasting condition* which ensures optimality of the choice of $V^{*}$, since if *F* were not continuous at $V^{*}$ then one could do better by investing at a different point (see Dixit and Pindyck (1994) for further discussion). We note that this is a *free-boundary problem* since the location of the boundary is unknown and must be determined as part of the solution. This threshold in the value of treatment can easily be converted to a threshold in the level of infected area ($I^{*}$) by dividing through by *p*.

**Table 1:** Form of the functions in the Bellman equation for each infection process *F*(*V*) must also satisfy the following boundary conditions

Stochastic process	$a\left( V \right)$	$b\left( V \right)$
Geometric Brownian motion	${\upbeta }V$	${\upsigma }V$
Mean-reverting SDE	${\upbeta }V\left( {1-\frac{V}{pI_{\mathrm{max}} }} \right)$	${\upsigma }V$
Logistic SDE	${\upbeta }V\left( {1-\frac{V}{pI_{\mathrm{max}} }} \right)$	${\upsigma }V\left( {1-\frac{V}{pI_{\mathrm{max}} }} \right)$

Due to the complexity of the logistic SDE, the problem no longer permits a closed-form solution and so the problem is solved numerically in MATLAB. Since the functions $a\left( V \right)$ and *b*(*V*) are non-linear for the logistic SDE, numerical solution of the ODE can be difficult. Therefore we instead find the long horizon limit of the associated finite time horizon problem using standard finite difference methods (Wilmott et al. 1995). See “Appendix” for further details. Baseline parameter values, and ranges for those parameters that are varied are given in Table 2. These parameters are based on those used in Ndeffo Mbah (2010).

**Table 2:** Parameter values used in numerical simulations

Model parameter	Description	Base case (range)
${\upbeta }$	Infection transmission rate	0.05 ([0.05, 0.8])
${\upsigma }$	Volatility	0.5 ([0.1, 0.9])
$I_{\mathrm{max}}$	Carrying capacity	100
*C*	Cost of treatment	20
*r*	Risk-free discount rate	0.1
*p*	Gain in yield per unit of infected area treated	1

## Results

**Fig. 1:**
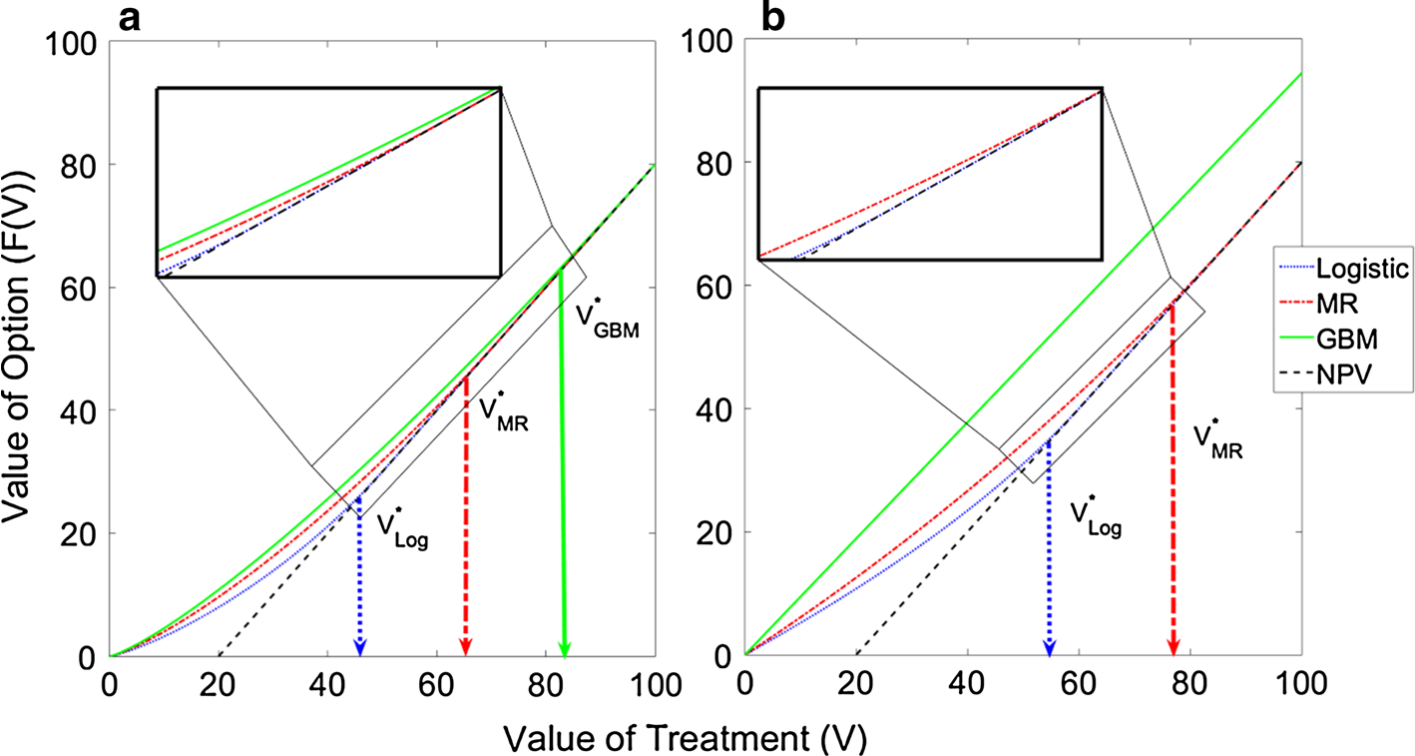
Value of the option to treat as a function of the value of treatment for the three different stochastic process assumptions. The standard NPV is shown as a *dashed line*. Plot **a** is for the case the where transmission rate is taken to be ${\upbeta }=0.05$ and the discount rate is taken to be $r=0.1$ and so ${\upbeta }<r$. Plot **b** is the case when ${\upbeta }=r=0.1$, and it can be seen in this case that GBM never intersects the standard NPV, showing it is never optimal to apply treatment. The volatility is taken to be ${\upsigma }=0.5$, and the other parameter values are given in Table 2

The solution to the free boundary problem associated with each SDE provides the value of the corresponding option to treat as a function of the treatment value (*V*). Figure 1 shows the value of this option as a function of the treatment value for the three different SDEs. Providing the value of the option to treat, $F\left( V \right)$, is greater than the NPV of immediate treatment, $F\left( V \right) >V-C$, there is value in retaining the option to treat, and so it is beneficial to wait. When $F\left( V \right) =V-C$, there is no additional gain in waiting and so treatment should be applied immediately. The value of treatment at which $F\left( V \right)$ first equals the NPV, the threshold value of treatment, $V^{*}$, is the boundary between the waiting region and the immediate treatment region and is also shown for each SDE in Fig. 1.

The threshold value of treatment, $$V^{*},$$ for each SDE corresponds to a threshold level of infection, $$I^{*}$$ ($$V^{*}=pI^{*})$$, at which treatment should be applied immediately. To provide a clearer illustration of situations when the optimal time to treat violates the natural upper boundary $$I^{*}\le I_{\mathrm{max}} $$, rather than the threshold value of treatment, $$\hbox {V}^*$$, we investigate how the threshold infection level at which treatment is applied *as a* *proportion* of the infected area, which we term the *threshold infected proportion*, $$I^{*}/I_{\mathrm{max}}$$, varies with different stochastic processes. We also explore the sensitivity of $$I^{*}/I_{\mathrm{max}}$$, to the epidemiological parameters $${\upsigma }$$ and $${\upbeta }$$ and discuss the implications for policy (note that, as in Sims and Finnoff (2013), we do not consider uncertainty over the economic parameters such as the price of agricultural commodity or timber). Finally, we consider the loss in value of the option to treat that arises from sub-optimally delaying treatment.

### Impact of Epidemiologically-Based Models on the Value of Treatment Threshold

**Fig. 2:**
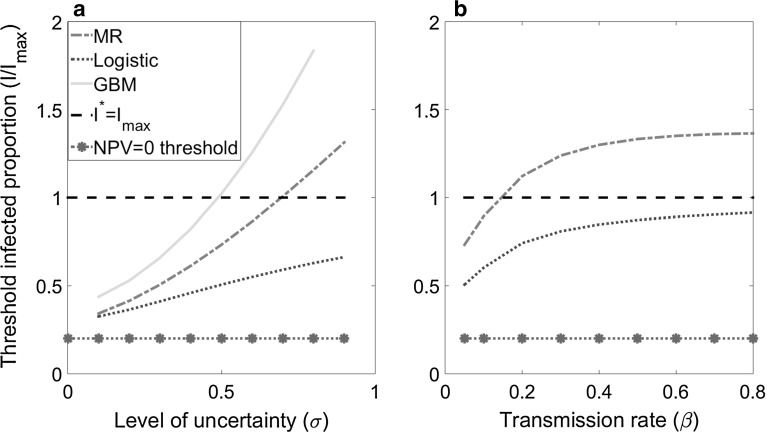
Threshold infected proportion ($I^{*}/I_{\mathrm{max}} )$: **a** as a function of volatility ($\sigma$) for all three stochastic processes and **b** as a function of the transmission rate (${\upbeta }$) for the mean-reverting and logistic SDEs only. The *dashed black line* shows when $I^{*}=I_{\mathrm{max}}$, and so it is clear that the mean-reverting and GBM can go above this natural boundary while this is not the case for the logistic SDE. Other parameter values are given in Table 2

Using an epidemiologically-based SDE to describe uncertainty in disease spread (mean-reverting or logistic SDE) decreases the threshold value and so treatment is deployed when a lower proportion of the area is infected, compared with the standard GBM assumption (Fig. 1). The effects hold for a wide range of parameter values (Fig. 2). As a result, the threshold at which treatment is applied is closer to the zero NPV level (shown as the purple dotted-stared line in Fig. 2) under the mean-reverting and logistic models than under GBM. As the level of infection becomes large, both the drift and diffusion terms for GBM are greater than for the other two SDEs. There is thus a greater probability that the value of treatment, *V*,  becomes large in the future which means the expected return from waiting is greater under the assumption of GBM. On the other hand, in the case of the logistic SDE as the level of infection approaches $I_{\mathrm{max}}$ the magnitude of both the drift and diffusion terms tend to zero and so the value of treatment cannot go above some maximal level $V_{\mathrm{max}} =pI_{\mathrm{max}}$, corresponding to the value of treatment when the whole area is infected ($I=I_{\mathrm{max}} )$. Hence there is a lower value to be obtained from waiting, and so it is optimal to apply treatment at a lower threshold level of infection (i.e. at a lower value of treatment). The threshold at which to act for the mean-reverting SDE lies between GBM and the logistic SDE. This reflects the trade-off between the growth in the drift term, which becomes dampened as the level of infection approaches $I_{\mathrm{max}}$, as for the logistic SDE, and the diffusion term which continues to increase with increasing levels of infection, as for GBM. Therefore the expected return from waiting is greater than for the logistic SDE but less than for GBM, and so the threshold value lies between the two.

The relative magnitudes of the infection rate (${\upbeta }$) and the discount rate *r* are important. If ${\upbeta }=r$, the option value under GBM never intersects the standard NPV (red dashed line) leading to the conclusion that treatment should never be applied. In fact, such a situation is equivalent to an American call in financial options and it has been shown that it is never optimal to exercise the option early, i.e. treatment should not be applied (Wilmott et al. 1995). This is typically why in previous studies the assumption is made that ${\upbeta }<r$ (Sims and Finnoff 2012) and indeed this is why we investigate the behaviour of GBM over a subset of the parameter values used for the other two processes. For the mean-reverting and logistic SDEs, a finite threshold value still exists if ${\upbeta }\ge r$ (Fig. 2). Therefore, using an epidemiological-based approach to modelling disease uncertainty ensures there are no restrictions on the range of transmission rate (${\upbeta }$) and discount rate (*r*) parameters investigated, unlike the case with GBM.

### Impact of Uncertainty and Transmission Rate on the Timing of Treatment and Value of Waiting

For all processes, the threshold value is a monotonic increasing function of volatility (Fig. 2a), which is consistent with options theory (Dixit and Pindyck 1994). The treatment threshold, $V^{*}$ (or $I^{*}$), represents the value of treatment (infection level) at which the benefit of immediate treatment, $V^{*}-C$, exactly equals the cost of immediate treatment in the form of the value of retaining the option to treat at a later date, which is forgone once treatment is applied. Increasing the volatility increases the option value of waiting by increasing the probability of more extreme outcomes including those where the net benefit of treatment is high, whilst leaving the value of immediate treatment unchanged, and thus increases the treatment threshold.

The rate of increase in the threshold infected proportion, $\left( {I^{*}/I_{\mathrm{max}} } \right) ,$ with increasing volatility (${\upsigma }$) is greater for GBM and the mean-reverting SDE than for the logistic equation (Fig. 2a). Therefore, the difference between the optimal time to treat between the three processes is greatest when uncertainty (${\upsigma }$) is large. Furthermore, the threshold infection level at which treatment is applied, $I^{*}$, increases above the maximum area that can be infected ($I_{\mathrm{max}} ),$ when ${\upsigma }>0.4$ for our base case parameters in the case of GBM and when ${\upsigma }>0.6$ in the case of the mean-reverting SDE. Hence, for large volatility, the threshold at which to apply treatment obtained from GBM or the mean-reverting equation is unattainable. Use of the GBM and mean-reverting models would therefore imply it would *never* be optimal to apply treatment for large volatility. This is not the case for the logistic model, where the threshold $I^{*}$ always remains below $I_{\mathrm{max}}$, and so is attainable (Fig. 2). We interpret this as follows. As volatility increases, the probability that the treatment value is large increases more for the mean-reverting or GBM-type SDE, whereas for the logistic SDE there is an upper bound to the value of treatment at $V_{\mathrm{max}} =pI_{\mathrm{max}}$ . Therefore, as volatility increases, the threshold at which to act remains bounded since the logistic SDE cannot reach large values above $V_{\mathrm{max}} =pI_{\mathrm{max}}$ in the future, no matter how large the volatility becomes.

Similarly, as the transmission rate increases, the threshold infected proportion at which to act increases for both the mean-reverting and logistic SDEs as shown in Fig. 2b. The threshold also increases for GBM since when ${\upbeta }\ge r$ the threshold is infinite. That is, as ${\upbeta }$ increases above the discount rate, the threshold at which to act increases to a level that is unattainable in finite time. Since we assume the value of treatment is proportional to the level of infection, treatment is most valuable when the level of infection is large. As the transmission rate increases, the level of infection is growing faster and so it is beneficial to wait longer. For very fast spreading diseases (${\upbeta }>0.2$ for our base case parameters) the threshold infection level $I^{*}$ for the mean-reverting SDE can go above $I_{\mathrm{max}}$, as with the case for large uncertainty (Fig. 2b). However, once again the logistic equation threshold remains within an attainable region (i.e. $I^{*}<I_{\mathrm{max}}$ ) (Fig. 2b). For large ${\upbeta }$, the threshold infected proportion levels off close to $I/I_{\mathrm{max}} =1$.

Since the primary aim of this paper is to investigate the impact of incorporating an epidemiological based model into the real options framework, we have focused on the sensitivity of the thresholds to the epidemiological important parameters, ${\upbeta }$ and ${\upsigma }$. However, we note that the model only depends on the combinations of the parameters ${\upbeta }/r$, ${\upsigma }^{2}/r$ and $C/\left( {pI_{\mathrm{max}} } \right)$. Therefore decreasing *r* has the same effect as increasing both ${\upbeta }$ and ${\upsigma }$, increasing threshold infection levels. In unreported simulations we find increasing the cost of control as a proportion of the maximum benefit from applying control, $C/\left( {pI_{\mathrm{max}} } \right)$, also increases thresholds for all three models, since the value of control needs to be greater in order to justify the additional costs. As above, when thresholds increase, particularly if $I^{*}/I_{\mathrm{max}} >1$ for GBM or the mean-reverting SDE, the difference between thresholds from different models also increases.

### Implications for Policy

**Fig. 3:**
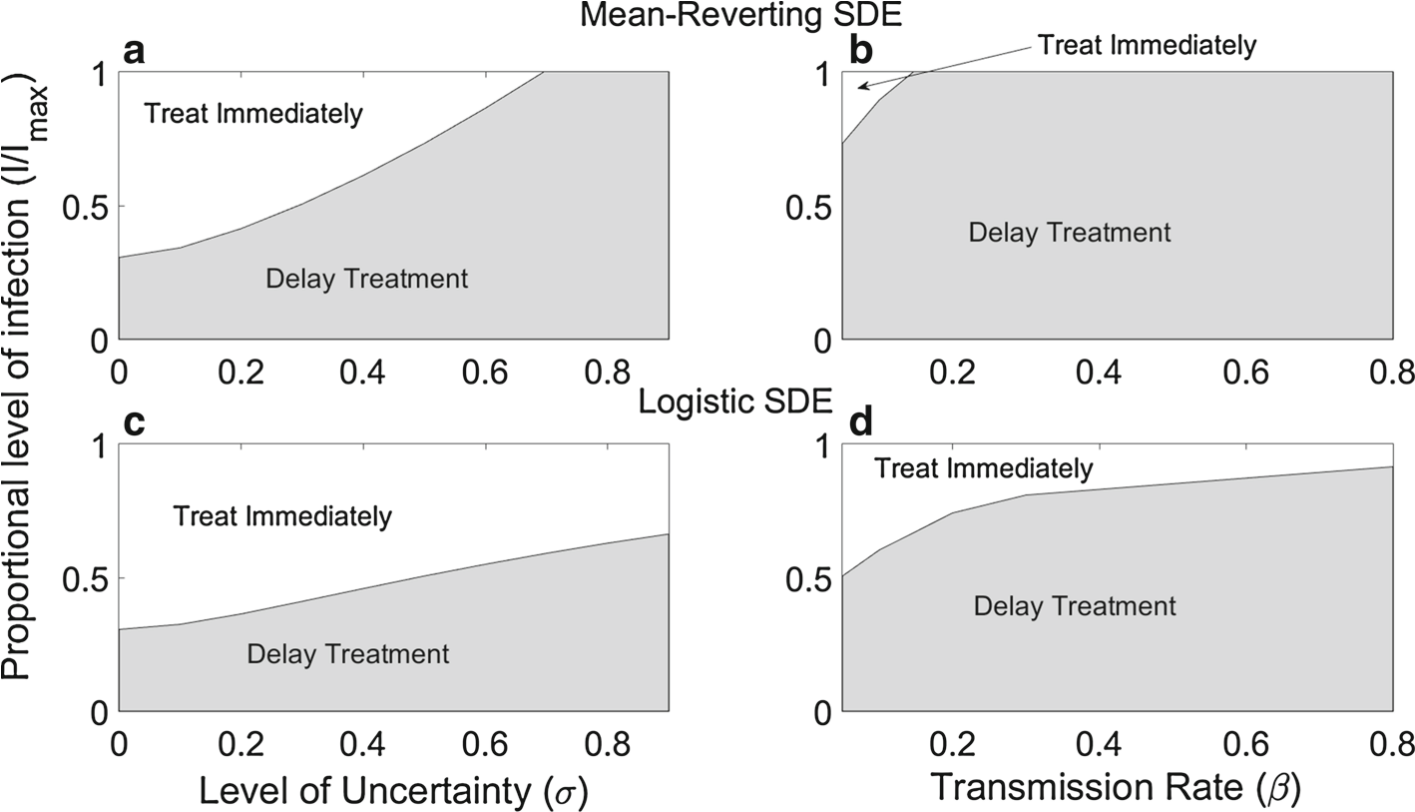
Policy plots showing the region in which treatment should be applied immediately (*unshaded region*) and where treatment should be delayed (*shaded region*) for the mean-reverting SDE (**a**, **b**) and the logistic SDE (**c**, **d**) for different levels of uncertainty (**a**, **c**) and transmission rates (**b**, **d**). Other parameter values are given in Table 2

The implications for decision makers of the different SDE models we consider can be seen in policy plots (Fig. 3). Under the logistic model, the waiting region is smallest, and so treatment will be applied earlier in the course of the epidemic, compared with assumptions based upon the GBM and the mean-reverting models. Furthermore, when there is great uncertainty (${\upsigma }$ large) or the disease is fast spreading (${\upbeta }$ large) there is no region in which treatment should be deployed under GBM or the mean-reverting equation, while under the logistic equation treatment should be applied when the proportion of infected area becomes large. Since the final size of the epidemic can be close to $I_{\mathrm{max}}$, not applying treatment could lead to large losses. Therefore, when uncertainty is large or the epidemic is fast spreading, the assumption of the logistic model is more appropriate than GBM or the mean-reverting equation since it provides a realistically attainable threshold at which to apply treatment.

**Fig. 4:**
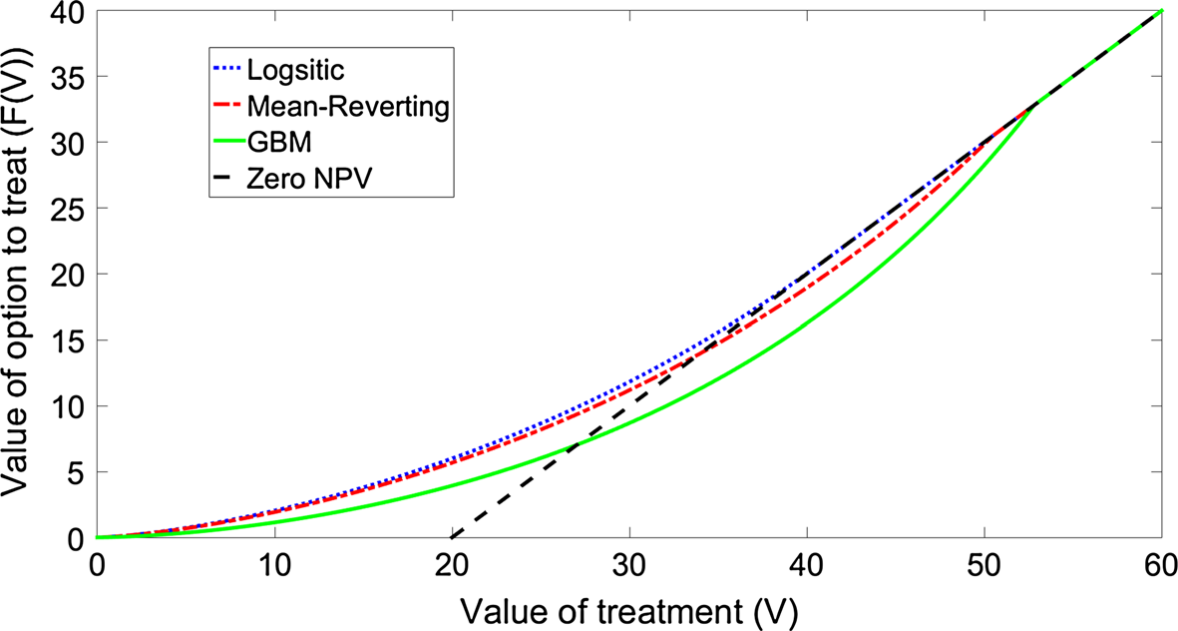
Value of the option to treat (*F*(*V*)) as a function of the treatment value (*V*) for the logistic model when treatment is applied at the wrong threshold, namely the threshold obtained from the mean-reverting model (*red dot-dashed line*) and the GBM model (*green solid line*). Also shown are the values of the option when treatment is applied at the optimal threshold (*blue dotted line*) and the standard NPV (*black dashed line*), i.e. the value of the option when NPV is zero. The volatility is taken to be ${\upsigma }=0.3$ and the transmission rate is taken to be $\upbeta =0.05$. (Color figure online)

**Fig. 5:**
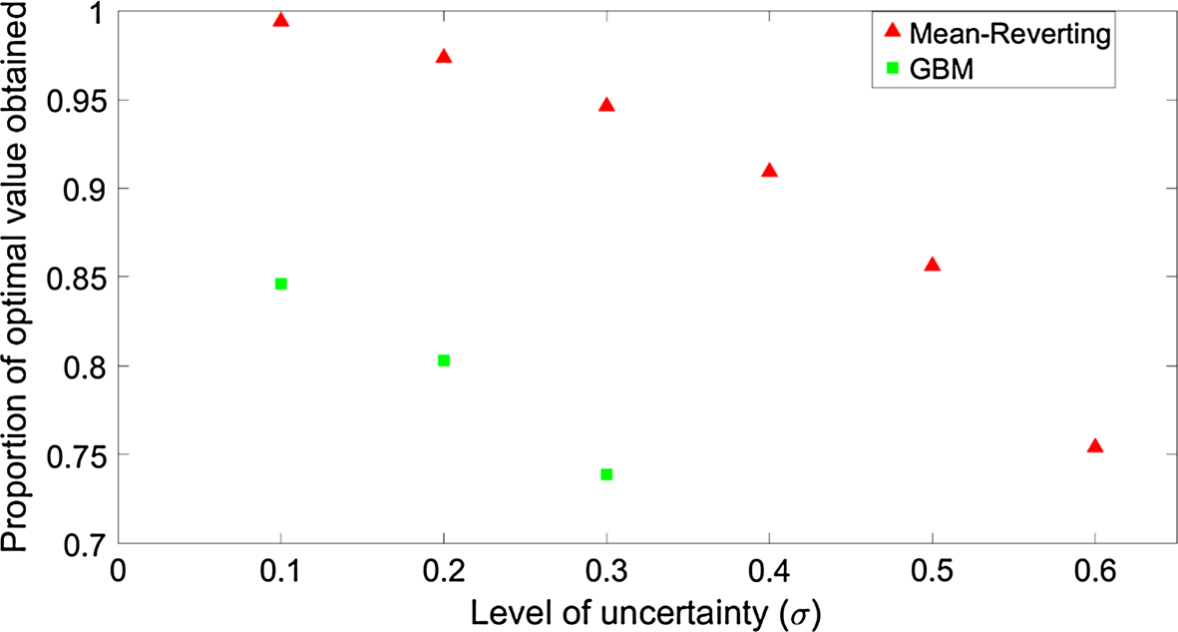
The proportion of the optimal value that is obtained under the logistic model when the treatment threshold from the mean-reverting (*red triangles*) and the GBM (*green squares*) models are enforced. The proportion of value obtained is shown as a function of the volatility (${\upsigma }$). (Color figure online)

### Loss in Value from Delaying Treatment Too Long: Consequences of the Wrong Threshold

To quantify the potential economic impact from inaccurate assumptions regarding the uncertainty in future disease spread, we consider the value of the option to treat if the evolution of infection follows the logistic equation but where the decision maker applies treatment at the ‘wrong’ threshold. This ‘wrong’ threshold is derived from either the mean-reverting or GBM model.^5^ Treating at the wrong threshold leads to a loss in value (Fig. 4). The loss arises because treatment thresholds are higher under mean-reversion or GBM than under the logistic assumption, and hence sub-optimally high when infection actually evolves according to the logistic model. This reduces the value of treating at a sub-optimally high threshold (Fig. 4, red and green lines), which fall below the standard NPV (Fig. 4, dashed black line). The loss in value is greatest for GBM, where a significant proportion of the optimal value (26$\%$ for our base case parameters) is lost by exercising control too late, i.e. at too high a threshold, while treating at the lower mean-reverting threshold leads to a lower loss of $5\% V$ for our base case parameters. Therefore, if the model used does not appropriately capture uncertainty in infection dynamics, then the excessive delay before treatment also implies that the full value of the option to treat is not realised. Figure 5 shows that the proportional loss from using the wrong assumptions increases with volatility for both the mean-reverting and GBM models. This is consistent with our earlier finding that the difference between the thresholds for the logistic and both the mean-reverting and GBM is greatest for large uncertainty. Also the proportional loss from using the mean-reverting thresholds is smaller than using the GBM thresholds since the mean-reverting threshold is closer to the threshold for the logistic equation (Fig. 1).

## Discussion and Conclusions

The real options approach has proved useful in investigating strategies for the optimal timing of measures to control disease and pest outbreaks. Uncertainty in disease spread is typically assumed to follow geometric Brownian motion due to its simplicity, rather than any epidemiological basis. However, ignoring the asymptotic boundary for infection and the deceleration in the rate of infection as this boundary is approached, may result in treatment being deployed too late, and indeed not at all if the level of uncertainty is high. This has significant implications for the formulation of the real options approach used to inform disease control policy.

We have shown that the stochastic process describing uncertainty in disease spread can be derived directly from basic epidemiological principles. Our principle results lead to the following conclusions:

1. When uncertainty is large or the epidemic is spreading fast, the threshold values for GBM and the mean-reverting model are unattainable, implying treatment should never be applied. This is not the case for the logistic model, where the threshold at which to treat always lies in a realistic range.
2. The threshold value at which to act is greatest for GBM and least for the logistic SDE. Therefore delaying treatment based on the results from the GBM or mean-reverting formulation may result in the application of treatment which is “too late”.
3. Applying treatment at the wrong threshold leads to a loss in value, and this loss will be greatest when uncertainty is large.
4. The differences in the threshold at which to treat between the three models increases with increasing volatility and transmission rate. Therefore the implications of the different models will be most disparate when there is great uncertainty (high $\upsigma$) and the infection is spreading rapidly (high $\upbeta$).

### Differences Between the Stochastic Processes and Consequences for the Timing of Control

Both the logistic and mean-reverting SDEs represent mechanistic models of future disease spread, and so, unlike GBM, the mean level of infection follows a logistic curve, which reflects the density-dependent nature of disease spread that is often observed experimentally. The impact of density-dependent growth is to reduce the value that can be obtained from treatment since there is a lower probability that the level of infection will become large, and thus the value obtained from treatment will be high. As a result GBM overvalues the option to treat, compared with the MR and logistic models and so leads to the greatest thresholds at which treatment should be adopted. Therefore, our results show that using an appropriate epidemiological-based model to describe the future uncertainty in disease spread is important since it alters the timing of control measures.

The key difference between the two epidemiological-based models studied in this paper, namely the logistic and the mean-reverting SDEs, is that the logistic SDE ensures trajectories respect the natural upper boundary of the system, namely the maximum number of hosts, while the mean-reverting SDE does not. This is because the diffusion coefficient in the mean-reverting SDE is the same as for GBM and so is unbounded, which can lead to individual trajectories violating the natural upper boundary of the system. On the otherhand, both the diffusion and drift coefficients of the logistic SDE are bounded, ensuring that the volatility as well as the mean rate of growth in the level of infection approaches its natural upper boundary. Since the value of treatment is higher the greater the level of infection, this means the mean-reverting SDE, as with GBM, over-estimates the value that can be obtained from treatment when the level of infection is high.

Since the logistic SDE captures both the logistic nature of the rate of infection, as well as the natural upper boundary of the system, it provides the most accurate description of future uncertainty in disease dynamics. When uncertainty is large or the disease fast spreading, the probability of the level of infection becoming large under the GBM and mean-reverting models is greatest. On the other-hand, due to the form of the drift and diffusion coefficients within the logistic model, the level of infection remains below the natural upper boundary, even when uncertainty is large or the disease fast spreading. The result is that in these regions of parameter space, the delay in treatment under the GBM or mean-reverting models will be greatest.

If treatment is excessively delayed, by using thresholds from the GBM or mean-reverting models when uncertainty in the level of infection is more appropriately described by the logistic SDE, only a portion of the optimal value is obtained. As uncertainty, and hence the difference between the thresholds, increases this proportion decreases, and so when uncertainty is high using predictions from the mean-reverting or GBM models leads to greater losses due to implementing treatment at the wrong threshold.

A further disadvantage of the GBM approach is that it restricts the range of parameter regimes that can be investigated since if ${\upbeta }\ge \hbox {r}$ the threshold value at which to apply treatment is not finite. Since the time horizon for forest management is long, typically 40–100 years, the discount rate used is usually taken to be lower than rates used for shorter-term projects^6^ (Pindyck 2006). Therefore, the assumption of GBM to characterise the uncertainty in the level of infection restricts application of the real options approach to slower spreading diseases.

Sims and Finnoff (2012) also examine the effects of incorporating an upper boundary on the spatial scale over which environmental damage (e.g. pests and pathogens) is measured. Rather than incorporating the natural upper boundary into the dynamics of future disease spread, Sims and Finnoff (2012) instead enforce the upper boundary directly whilst continuing to assume the future level of damage evolves according to GBM. Incorporating spatial scale in this way ensures the threshold at which control is first adopted lies below the upper boundary; however for certain parameter combinations Sims and Finnoff (2012) find that control is never optimal. A direct comparison (incorporating their framework into our model) is not possible because their approach incorporates the upper boundary in future disease spread through the *payoff *to control, which depends on the (constrained) future level of damages. In contrast, in order to focus on the effects of different SDEs, we assume a particularly simple payoff function which is not forward-looking i.e. it depends only on the level of infection when control occurs. Understanding the similarities and differences between the two approaches, including when the closed-form thresholds from Sims and Finnoff (2012) are close to those from an epidemiological-based model which requires numerical solution, would require a more complex and realistic model which we leave for further work. However, we expect that, since the Sims and Finnoff (2012) approach does not capture the density-dependent nature of the evolution of the level of infection over time, the two approaches should give closer results when epidemiological constraints have less effect, e.g. at the early stages of an infection and when the spatial scale of the decision-maker represents a relatively small portion of the total potentially infected area.

### Future Work

We have shown how to incorporate an epidemiological modelling framework into a real options approach to study the optimal timing of disease treatment in the presence of uncertainty. In particular, since the main aim of this paper is to investigate the effect of more realistic characterisations of disease uncertainty on the conclusions of the real options model, we have used a simple formulation of the decision problem. Future work could examine the interaction of an epidemiologically-based SDE for the evolution in the level of infection with more complex models of costs and impacts of different control strategies. For example, for diseases for which there is no treatment available that will eradicate infection, the additional sunk benefit associated with early application of treatment due to the reduction in potential future damage as a result of disease could be incorporated as in Dixit and Pindyck (1994, pp. 412–418) and Sims and Finnoff (2012). Also economic uncertainty (market risk), which is potentially correlated with disease risk, as well as the interaction effects between economic and ecological uncertainty, could be included into the decision problem as proposed in Sims and Finnoff (2013). Finally the model presented here is not explicitly spatial, so although we have some notion of maximum spatial size (e.g. the maximum number of trees), we essentially homogenise over space. The epidemiological model presented here could be extended to incorporate space explicitly, to allow the potential effects of re-infection on the optimal timing of treatment to be investigated. We leave these aspects for future work.

The approach described here provides a convenient method for formulating the future uncertainty since it is derived directly from epidemiological principles of pathogen transmission. Furthermore, this framework can be extended to more complicated epidemiological-based models of disease spread that capture additional features specific to a particular pathogen/pest combination such as cryptic infection (hosts become infectious before showing symptoms) (Gilligan 2008; Cunniffe et al. 2016) or which allow for external infection pressure (Ndeffo Mbah and Gilligan 2010). Furthermore, the best way in which to incorporate uncertainty into the deterministic formulation of epidemiological models is an area of active investigation, and there are a number of different approaches that can be taken (Keeling and Rohani 2008; Allen 2007). The approach taken here is to include a random perturbation to the transmission parameter since this is where the main source of uncertainty lies. Future work could consider the impact of different approaches to incorporating uncertainty into the deterministic epidemiological models. For example, one could assume that the transmission parameter itself is described by a stochastic process, such as an Ornstein–Uhlenbeck process (Allen 2007); or by considering additional sources of uncertainty such as demographic stochasticity.

One important issue with using the real options framework to assess the impact of uncertainty on the optimal timing of disease control measures is that the form of the process that best represents uncertainty in disease spread is rarely discussed. Here we have shown that the form of this process can have a significant impact on the conclusions of the real options model, with different models for the uncertainty in disease spread leading to contrasting implications for decision makers.

## Appendix

### Formulation of the Bellman Equation

The evolution in the value of treatment is given by the SDE,

$$\begin{aligned} \hbox {d}V=a\left( V \right) \hbox {d}t+b\left( V \right) \hbox {d}W, \end{aligned}$$

where the functions $\hbox {a}\left( V \right)$ and $\hbox {b}\left( V \right)$ differ depending of the stochastic process assumed (see Table 1 in the main text).

At any point in time $\hbox {t}$, the decision authority either applies treatment (i.e. invests in treatment) and obtains the net gain $V\left( t \right) -C$, or waits and obtains the expected change in the value of the option. Therefore, we can formulate the decision of whether or not to apply treatment as an optimal stopping problem, described by the following

$$\begin{aligned} F\left( {V,t} \right) =\hbox {max}[\left( {V-C} \right) ,\hbox {e}^{-r\hbox { d}t}\mathbb {E}[F\left( {V+\hbox {d}V,\,t+\hbox {d}t} \right) |V]]. \end{aligned}$$

The first term on the right is the value the decision authority would obtain if treatment were applied immediately while the second term is the expected value in retaining the option to treat in the future, discounted at rate *r*. Therefore, in the region where it is optimal to wait and not treat immediately, referred to as the *continuation region*, the second term on the right hand side of the equation will be larger. It follows that in the continuation region

$$\begin{aligned} r\hbox {F}\left( {V,t}\, \right) \hbox {d}t=\mathbb {E}\left[ {\hbox {d}F} \right] . \end{aligned}$$

Since $V\left( t \right)$ is a stochastic process, then using Ito’s Lemma we can evaluate $\hbox {d}F$ as follows,

$$\begin{aligned} \hbox {d}F=\frac{\partial F}{\partial t}+\frac{1}{2}\frac{\partial ^{2}F}{\partial V^{2}}\left( {\hbox {d}V} \right) ^{2}+\frac{\partial F}{\partial V}\hbox {d}V. \end{aligned}$$

Substituting in the equation for $\hbox {dV}$, and noting that $\mathbb {E}\left[ {\hbox {dW}} \right] =0$, we obtain the following

$$\begin{aligned} \mathbb {E}\left[ {\hbox {d}F} \right] =\frac{\partial F}{\partial t}\hbox {d}t+\frac{1}{2}b\left( V \right) ^{2}\frac{\partial ^{2}F}{\partial V^{2}}\hbox {d}t+a\left( V \right) \frac{\partial F}{\partial V}\hbox {d}t, \end{aligned}$$

where $a\left( V \right)$ and $b\left( V \right)$ are the drift and diffusion coefficients, respectively, of $V\left( t \right)$ (given in Table 1 in the main text).

Using the fact that $rF\hbox {d}t=\mathbb {E}\left[ {\hbox {d}F} \right]$, we arrive at the following partial differential equation (PDE)

$$\begin{aligned} \frac{\partial F}{\partial t}+\frac{1}{2}b\left( V \right) ^{2}\frac{\partial ^{2}F}{\partial V^{2}}+a\left( V \right) \frac{\partial F}{\partial V}-rF=0. \end{aligned}$$

Whilst, in principle, the value of the option to treat can be a function of time, *t*, as well as the current value of treatment, *V*, since there is no terminal date beyond which treatment cannot be applied the payoff is independent of time and the problem is time invariant, so $\frac{\partial F}{\partial t}=0$ and *F* is the solution of the ODE (6):

$$\begin{aligned} \frac{1}{2}b\left( V \right) ^{2}\frac{\hbox {d}^{2}F}{\hbox {d}V^{2}}+a\left( V \right) \frac{\hbox {d}F}{\hbox {d}V}-rF=0, \end{aligned}$$

subject to the following boundary conditions

$$\begin{aligned}&\displaystyle F\left( {0,\, t} \right) =0\\&\displaystyle F\left( {V^{*},t} \right) =V^{*}-C\\&\displaystyle \frac{\hbox {d}F}{\hbox {d}V}\left( {V^{*},\,t} \right) =1. \end{aligned}$$

### Solution Method

The problem described in Sect. 3.3 assumes that the decision maker can apply treatment at any point in the future, i.e. the time horizon for the problem is infinite. We solve Eq. (6) (see main text) and associated boundary conditions by finding the long horizon limit of the associated finite horizon problem

A1 $$\begin{aligned} \frac{\partial F}{\partial t}+\frac{1}{2}b\left( V \right) ^{2}\frac{\partial ^{2}F}{\partial V^{2}}+a\left( V \right) \frac{\partial F}{\partial V}-rF=0, \end{aligned}$$

such that $F\left( {0,\,t} \right) =0$, $F\left( {V^{*}\left( t \right) ,t} \right) =V^{*}\left( t \right) -C$, $\frac{\partial F}{\partial V}\left( {V^{*}\left( t \right) ,\,t} \right) =1$, $F\left( {V,T} \right) =\hbox {max}\left( {0,V-C} \right)$, which assumes treatment can only be applied until time *T*, so both the differential equation and the threshold value, $V^{*}\left( t \right)$ depend explicitly on time. This free boundary problem can be solved straightforwardly using the Crank–Nicolson finite-difference method with successive-over-relaxation (SOR) (Wilmott et al. 1995).

In order to solve the problem numerically, an upper boundary condition must be stipulated in the case where it is not worthwhile treating immediately. We assume that as *V* becomes large $\partial ^{2}F/\partial V^{2}=0$ which is used to obtain the finite difference scheme at the upper boundary (Insley 2002).

We take *T* sufficiently large that $\frac{\partial F}{\partial t}\rightarrow 0$. We find that taking $T=100$ is more than a sufficient length of time to ensure steady state is reached for all three stochastic processes.

The values of model parameters and parameters within the numerical method used in simulations are given in Table 2 in the main text.

### Exercising the option at the wrong threshold

In order to determine the value of the option to treat if the wrong threshold is implemented in the logistic model we solve the PDE given by (A1) with *a*(*V*) and *b*(*V*) taken from the logistic SDE, again using the Crank–Nicolson finite-difference scheme. However, we no longer have a free boundary problem, since we know the upper boundary at the threshold value (where the threshold value is taken from that obtained for the mean-reverting or GBM model). Let $V^{\mathrm{W}}$ be the threshold value obtained from the mean-reverting or GBM model. The boundary conditions for (A1) are thus

$$\begin{aligned} F\left( 0 \right) =0\hbox { and }F\left( {V^{\mathrm{W}}} \right) =V^{\mathrm{W}}-C. \end{aligned}$$

## Footnotes

1
http://www.forestry.gov.uk/fr/rootandbuttrot.

2
http://www.forestry.gov.uk/pdf/RIN224.pdf/$FILE/RIN224.pdf.

3
http://www.forestry.gov.uk/fr/rootandbuttrot.

4
http://www.forestry.gov.uk/pdf/RIN224.pdf/$FILE/RIN224.pdf.

5 This is calculated by solving the differential equation associated with the logistic equation but imposing the threshold values obtained from either the mean-reverting or the GBM models (see “Appendix” for details).

6 What the appropriate discount rate should be is itself a frequently discussed topic (Gollier 2011), with previous studies using discount rate values of 8% (Sims and Finnoff 2012) and 10% (Ndeffo Mbah 2010). For many years, the UK Forestry Commission used a rate much lower than the standard public sector test rate of discount, partly to take into account the longer time horizons in forest investment (Hanley and Spash 1993).
